# Central Nervous System Involvement by Novel Clade 2.3.2.1e H5N1 Avian Influenza Virus in a Pediatric Patient

**DOI:** 10.1093/ofid/ofag283

**Published:** 2026-05-07

**Authors:** Phung Nguyen The Nguyen, Nguyen Thanh Hung, Ngo Ngoc Quang Minh, Nguyen Thi Thu Hong, Nguyen Thi Thanh Huong, Cao Minh Hiep, Le Nguyen Thanh Nhan, Tran Van Dinh, Du Tuan Quy, Tran Thanh Thuc, Tran Minh Nhut, Nguyen Thi Han Ny, Lam Anh Nguyet, Le Nguyen Truc Nhu, Do Duong Kim Han, Truong Hoang Chau Truc, Le Thi Tam Uyen, Nghiem My Ngoc, Tran Nguyen Phuong Thao, Tran Thi Thanh Tam, Sandy Tze-Minn Mak, Jurre Y Siegers, Sebastian Maurer-Stroh, Nguyen Thanh Dung, Erik A Karlsson, Guy Thwaites, Chee Wah Tan, Nguyen Van Vinh Chau, Le Van Tan, SEACOVARIANTS and H5N1 Consortia, Nguyen To Anh, Nguyen Thi Thu Hong, Truong Hoang Chau Truc, Nguyen Thi Han Ny, Do Duong Kim Han, Le Kim Thanh, Lam Anh Nguyet, Cao Thu Thuy, Le Nguyen Truc Nhu, Tran Tan Thanh, Lam Minh Yen, Vu Thi Ty Hang, Pham Ngoc Long, Pham Tieu Kieu, Vo Tan Hoang, Nguyen Thi Thao, Mary Chambers, Vu Duy Thanh, Pham Ngoc Long, H Rogier van Doorn, Trinh Son Tung, C Louise Thwaites, Guy Thwaites, Lin-Fa Wang, Beng Lee Lim, Raph L Hamers, Anuraj Shankar, Suwatrti Suwarti, Tayipto Tayipto, Eva Simarmata, Ragil Dien, Juthathip Mongkolsapaya, Wanwisa Dejnirattisai, Warangkana Chantima, Narisara Chantratita, Prapassorn Poolchanuan, Vichapon Tiacharoen, Adul Dulsuk, Sophon Iamsirithaworn, Nick Day, Phaik Yeong Cheah, Tassawan Poomchaichote, Kanpong Boonthaworn, Gavin Screaton, Aiete Dijokaite-Guraliuc, Raksha Das, Chang Liu, Piyada Supasa, Muneeswaran Selvaraj, Susanna J Dunachie, Paul Klenerman, E Yvonne Jones, David I Stuart, Barbara Kronsteiner-Dobramysl, Martha Zewdie, Priyanka Abraham, Jennifer Hill, Nghiem My Ngoc, Alba Grifoni, Alessandro Sette, Wee Chee Yap, Chee Wah Tan, Le Van Tan

**Affiliations:** University of Medicine and Pharmacy at Ho Chi Minh City, Ho Chi Minh City, Vietnam; Children's Hospital 1, Ho Chi Minh City, Vietnam; Children's Hospital 1, Ho Chi Minh City, Vietnam; Children's Hospital 1, Ho Chi Minh City, Vietnam; Oxford University Clinical Research Unit, Ho Chi Minh City, Vietnam; Children's Hospital 1, Ho Chi Minh City, Vietnam; Children's Hospital 1, Ho Chi Minh City, Vietnam; Children's Hospital 1, Ho Chi Minh City, Vietnam; Children's Hospital 1, Ho Chi Minh City, Vietnam; Children's Hospital 1, Ho Chi Minh City, Vietnam; University of Medicine and Pharmacy at Ho Chi Minh City, Ho Chi Minh City, Vietnam; Children's Hospital 1, Ho Chi Minh City, Vietnam; Children's Hospital 1, Ho Chi Minh City, Vietnam; Oxford University Clinical Research Unit, Ho Chi Minh City, Vietnam; University of Medicine and Pharmacy at Ho Chi Minh City, Ho Chi Minh City, Vietnam; Oxford University Clinical Research Unit, Ho Chi Minh City, Vietnam; Oxford University Clinical Research Unit, Ho Chi Minh City, Vietnam; Oxford University Clinical Research Unit, Ho Chi Minh City, Vietnam; Oxford University Clinical Research Unit, Ho Chi Minh City, Vietnam; Hospital for Tropical Diseases, Ho Chi Minh City, Vietnam; Hospital for Tropical Diseases, Ho Chi Minh City, Vietnam; Hospital for Tropical Diseases, Ho Chi Minh City, Vietnam; Hospital for Tropical Diseases, Ho Chi Minh City, Vietnam; A*STAR Bioinformatics Institute, Agency for Science, Technology and Research, Singapore, Singapore; Institut Pasteur du Cambodge, Cambodia; A*STAR Bioinformatics Institute, Agency for Science, Technology and Research, Singapore, Singapore; Yong Loo Lin School of Medicine and Department of Biological Sciences, National University of Singapore, Singapore, Singapore; A*STAR Bioinformatics Institute, Agency for Science, Technology and Research, Singapore, Singapore; Institut Pasteur du Cambodge, Cambodia; Oxford University Clinical Research Unit, Ho Chi Minh City, Vietnam; Nuffield Department of Medicine, University of Oxford, Oxford, United Kingdom; Infectious Diseases Translational Research Programme, Department of Microbiology and Immunology, Yong Loo Lin School of Medicine, National University of Singapore, Singapore, Singapore; Department of Health, Ho Chi Minh City, Vietnam; Oxford University Clinical Research Unit, Ho Chi Minh City, Vietnam; Nuffield Department of Medicine, University of Oxford, Oxford, United Kingdom

**Keywords:** H5N1, highly pathogenic avian influenza, meningoecephalitis, Vietnam

## Abstract

Novel clade 2.3.2.1e A(H5N1) virus was detected in cerebrospinal fluid but not in respiratory, rectal swab, or blood samples of an 8-year-old boy presenting with meningoencephalitis without respiratory symptoms. Cerebrospinal fluid A(H5N1) hemagglutinin–specific antibody levels were higher than those of sera. Clinicians should be aware of emerging clade 2.3.2.1e A(H5N1)–associated meningoencephalitis.

Highly pathogenic avian influenza (HPAI) A(H5N1) virus is a public health threat with pandemic potential. H5N1 infection in humans can result in severe respiratory disease but is rarely accompanied by central nervous system (CNS) involvement. Herein, we report on HPAI A(H5N1)-associated meningoencephalitis in the absence of respiratory symptoms in an 8-year-old boy.

## PATIENT PRESENTATION AND INVESTIGATIONS

In mid-April 2025, a previously healthy 8-year-old boy from Tay Ninh province in Vietnam, bordering Cambodia, was admitted to a local hospital with a 24-hour history of fever, severe headache, and vomiting, without any respiratory symptoms. Routine blood tests showed leukocytosis (23 700 cells/μL) and mildly elevated platelet level (456 000 platelets/μL) (Supplementary Table 1). Rapid NS1 test for dengue virus was negative. He was diagnosed with sepsis/meningoencephalitis. Two days later he was transferred to the Children's Hospital 1 (CH1), a tertiary referral hospital in Ho Chi Minh City. At CH1, he presented with fever (38.1°C) and neck stiffness with altered consciousness, but without apparent respiratory illness. His chest radiograph showed consolidation, corresponding to the interpretation of left lower lobe findings (Supplementary Figure 1). Chest computed tomography was not performed. However, he had no respiratory symptoms and had normal heart and respiratory rates (95 beats per minute and 22 breaths per minute, respectively), normal oxygen saturation on room air (95%), and normal auscultation of the lungs. The admission diagnosis was suspected meningoencephalitis. Empiric intravenous ceftriaxone, vancomycin, and acyclovir were started on the basis of clinical presentations.

**Figure 1.**
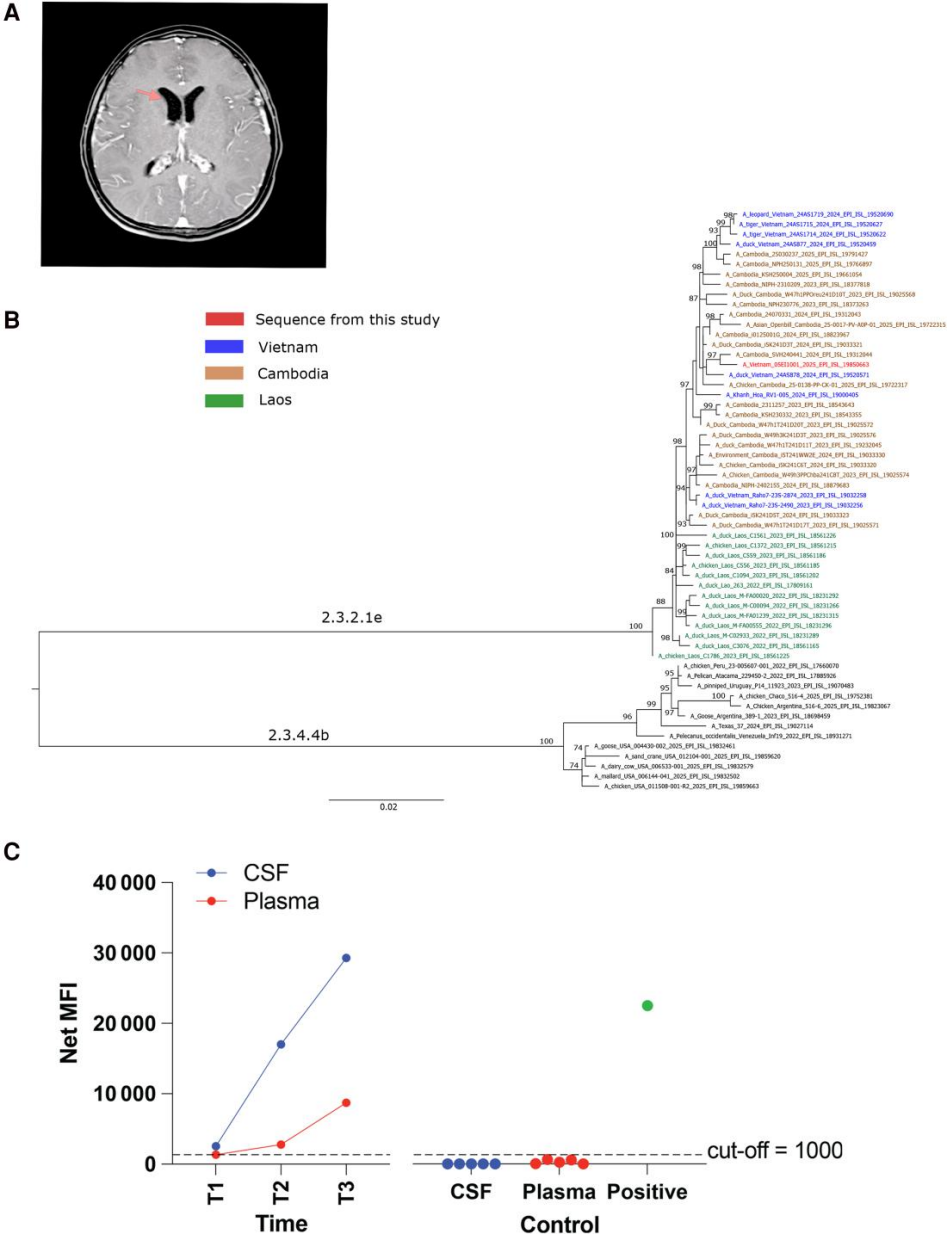
Results of brain image and laboratory investigations. *A*, T2 fluid-attenuated inversion recovery sequence after contrast administration of brain magnetic resonance imaging obtained on illness day 6 showing dilated lateral ventricles with a dimension of 14 mm, marked by red arrow (normal range: ≤10 mm). *B*, Reconstructed maximum likelihood (ML) trees of hemagglutinin-segment sequences obtained from this study and representatives of the corresponding gene segment sequences of clades 2.3.2.1e and 2.3.4.4b. *C*, Antibodies against HA1 subunit in serial CSF and plasma samples collected over the course of illness. In (*C*), the dashed line indicates assay cut-off. Negative control samples include 5 CSF samples from patients with central nervous system infection (bacterial meningitis due to *Enterococcus faecium* [n = 1], tuberculous meningitis [n = 2], cryptococcal meningitis [n = 1], and cerebral tumor [n = 1]), and 5 plasma samples from healthcare workers participating in a COVID-19 vaccine evaluation study (Supplementary Appendix). Positive control was derived from plasma sample of a patient with polymerase chain reaction–confirmed H5N1 infection. T1, T2, and T3 for CSF are samples collected on illness day 6, 12, and 18, respectively. T1, T2, and T3 for plasma are samples collected on illness day 6, 11, and 21, respectively. Abbreviations: CSF, cerebrospinal fluid; MFI, mean fluorescence intensity.

Cerebrospinal fluid (CSF) collected on admission (day 3 of illness) showed pleocytosis (486 cells/μL) with neutrophil predominance, elevated lactate and protein concentrations, and hypoglycorrhachia (Table 1). Blood hematologic indices were within normal ranges (Supplementary Table 1). Brain magnetic resonance imaging (MRI) revealed dilated lateral ventricles (Figure 1*A*). Routine bacterial culture of admission CSF, urine, blood, and endotracheal aspirate samples was negative, but analysis of the admission CSF, using a multiplex real-time reverse-transcription polymerase chain reaction (PCR) platform targeting >60 pathogens [1], revealed influenza A virus (IAV) with a cycle threshold (Ct) value of 19. Subsequent confirmatory IAV and subtyping PCR testing of a second CSF sample collected on day 6 of illness onset using US Centers for Disease Control and Prevention assays returned IAV (Ct = 26) and A/H5 (Ct = 34, Supplementary Tables 2 and 3). PCR testing for IAV in urine, throat swabs, rectal swabs, endotracheal aspirate, and blood samples was all negative, while serial CSF samples collected until day 10 of hospitalization were all positive (Supplementary Table 3). Details about the commercially available diagnostic assays and the targeted pathogens are presented in the Supplementary Appendix and the Supplementary Table 3 footnote.

**Table 1.** Laboratory Findings of Admission and Follow-up Cerebrospinal Fluid Samples

Laboratory Examination	Normal Range	CSF1 (Admission), Illness Day 3	CSF2, Illness Day 6	CSF3, Illness Day 8	CSF4, Illness Day 12	CSF5, Illness Day 18
Leukocyte count, cells/μL	≤5	486	418	550	118	160
Neutrophils, %	0	77	85	78	83	57
CSF glucose concentration, mmol/L	2.8–4.4	1.1	2.1	1.8	2.9	1.9
CSF/plasma glucose ratio, %	≥0.6	0.16	0.23	0.3	0.57	0.3
Lactate, mmol/L	1–2	10.4	8.7	4.9	2.73	3.29
Protein, g/L	<0.4	3.6	11.7	6.2	2.7	3.06

Abbreviation: CSF, cerebrospinal fluid.

After A/H5 PCR results became available, epidemiological investigations were initiated and revealed that his family owned many young fighting cocks, which the patient treated as pets, with frequent close contact. Around 2 weeks before his illness, some sporadically died of unknown causes. The patient, however, did not have any respiratory symptoms within the 2 weeks preceding his present illness. The neighbors also reported that groups of 40–50 chickens died of unknown reasons at around the same time. No poultry samples were available for testing.

Mechanical ventilation was initiated 15 hours after admission to CH1 because of worsening coma and suspected cerebral edema. Subsequently, the patient's condition deteriorated, progressing to a deep coma with no response to stimuli and unstable hemodynamic observations, requiring vasopressor and inotropic support. Intravenous mannitol and hypertonic saline were administered to reduce elevated intracranial pressure. Based on the PCR results, MRI findings, and clinical features, the patient was diagnosed with meningoencephalitis due to IAV A(H5N1) virus infection and isolated in accordance with local public health measures. Acyclovir treatment was discontinued, and oseltamivir was commenced on day 3 of hospitalization, followed by intravenous immunoglobulin. Vancomycin was changed to linezolid, and levofloxacin was added on the basis of recurrent fever and elevated procalcitonin level (Table 1). Durations of antiviral and antibiotic administration are detailed in Supplementary Table 3. The patient recovered and was extubated on day 8 of hospitalization. Follow-up CSF remained abnormal until day 18 of illness (Table 1), when IAV RNA in the CSF was undetectable (Supplementary Table 4). As part of our routine care, obtaining clinical samples (especially CSF) for routine diagnosis was verbally agreed by the parents, and was based on the basis of clinical progression and local public health measures requiring that the patient tested negative before discharge. He was discharged with full recovery after 19 days of hospitalization.

## WHOLE GENOME SEQUENCING AND SEQUENCE ANALYSIS

Direct sequencing of the second CSF sample recovered all 8 gene segments of the IAV genome. Laboratory workflow is detailed in the Supplementary Appendix. Phylogenetic analysis assigned the hemagglutinin (HA) gene segment to clade 2.3.2.1e (Figure 1*B*, Supplementary Figures 2 and 3, Supplementary Table 5), previously known as clade 2.3.2.1c of HPAI A(H5N1) viruses that circulate endemically in Southeast Asia. More specifically, the obtained sequences belonged to a novel reassortant clade 2.3.2.1e of HPAI A(H5N1) with gene segments coming from both clade 2.3.2.1c and clade 2.3.4.4b viruses [2] that emerged in late 2023, causing outbreaks in poultry and zoonotic infections in mammals, including 14 confirmed human cases (6 deaths) in Cambodia and 1 human case (fatal) in Vietnam. Likewise, all remaining segments were closely related to the corresponding segments of clade 2.3.2.1e reassortant genotype viruses (Supplementary Figure 3). Further in-depth phylogenetic and phylogeographic analysis is beyond the scope of this study and was hindered by a lack of contemporary sequences, especially from poultry in Vietnam. However, the placement of gene segments on the corresponding phylogenetic trees suggested that the viral strain is closely related to sequences recovered from recent human cases and poultry samples in Vietnam and Cambodia [2]. Several amino acid substitutions associated with the host specificity shift and mammalian adaptation were observed (Supplementary Table 6). Of these, 2 substitutions in the HA sequences, S123P and R167K, associated with increased binding of the virus to α2,6 receptors, were unique to the virus of the present study (Supplementary Table 6). Otherwise, the remaining mutations have previously been reported.

Using microsphere immunoassay, immunoglobulin G antibodies against H5 A/Cambodia/i0125001G/2024) HA1 subunit were detectable in plasma and CSF samples collected at day 11–12 of illness, with CSF antibody levels higher than those of plasma (Figure 1*C*). Specifically, the mean fluorescence intensity value increased from a borderline level to 2542 and 29 286 in the CSF as compared to from an undetectable level to 1363 and 8723 in the plasma. Anti-H5 hemagglutinin antibody was not detectable in negative control CSF and serum samples (Figure 1*C*).

For both parents, their throat swabs and plasma samples collected at enrollment were negative for IAV by PCR analysis. Likewise, their plasma samples were also negative for antibodies against the HA1 subunit (data not shown).

## DISCUSSION

Influenza A(H5N1)–associated CNS infection in humans has rarely been reported but typically presents as a complication, following respiratory symptoms [3–5]. Notably, our patient presented with meningoencephalitis in the absence of respiratory symptoms. Additionally, unlike the previously reported patients, who had viral RNA detected in both CSF and non-CSF samples [3–5], our patient only had viral RNA detected in serial CSF samples in the absence of viral RNA detected in urine, blood, rectal swab, and respiratory samples. Low respiratory tract viral loads, transient viral replication in the respiratory tract, and/or delayed sample collection (illness day 6 onward) might explain the negative PCR findings in non-CSF samples, including the endotracheal aspirate sample. Notably, HPAI A(H5N1) viruses can infect human respiratory tissues by binding to receptors bearing sialic acids linked to galactose by α2,3-linkages, which are found in the lungs and lower respiratory tract, supported by the chest radiograph findings suggestive of lower left lung pneumonia.

Intrathecal antibody production following seasonal influenza virus infection has been reported previously [6, 7]. Likewise, we showed that the titers of antibodies against A(H5N1) HA were higher in the CSF than in the plasma. This suggested that CSF antibodies were likely intrathecally produced as a consequence of viral invasion of the CNS, which can occur via hematogenous pathway by passing the blood–brain barrier, or via cranial nerves, especially the olfactory nerve route without viremia [3, 8, 9]. Based on our collective findings, it is likely that the A(H5N1) virus from poultry entered the CNS without establishing a significant infection phase in epithelial cells of the nasal cavity [10]. Therefore, future study should assess the mucosal immune response to A(H5N1) to further shed light on the disease pathogenesis.

The CSF findings of our patient showed neutrophil predominance and hypoglycorrhachia, which were more compatible with bacterial meningitis but inconsistent with findings from previous reports about human cases of A(H5N1) virus–associated CNS infection [4, 5, 11–15]. Head and sinus computed tomography, however, was not performed. Therefore, although routine culture and PCR were negative for common bacterial causes, we cannot exclude other possibilities (eg, parameningeal infections).

Mammalian-adapted mutations have been documented in some clade 2.3.4.4b viruses causing outbreaks in cows in the United States [16]. Likewise, we documented 2 substitutions (S123P and R167K) in the HA sequences associated with increased virus binding to α2,6 receptors that are unique to the virus of the present study. These data emphasize the increasing risk of A(H5N1) virus adapting to mammals and becoming more neurologically virulent and more transmissible. However, molecular and serological testing of 180 households of human cases in the United States was negative [16], demonstrating the absence of human-to-human transmission, supporting our findings.

Our study has some limitations. First, the microsphere immunoassay used in this study is an investigational assay. Therefore, the detection of CSF antibodies should be further validated using gold standard tests such as hemagglutination inhibition or live-virus neutralization assay. Second, epidemiological investigations were only carried out after HPAI A(H5N1) PCR results became available, which resulted in a delayed oseltamivir administration. Clinicians in endemic regions should be aware of meningoencephalitis associated with A(H5N1) infection.

In summary, we report on an HPAI A(H5N1) infection in a child presenting with meningoencephalitis in the absence of respiratory symptoms. Viral RNA was detected in CSF but not in respiratory, rectal swab, or blood samples. Testing for IAV and A(H5N1) virus should be considered in patients presenting with CNS infection with a history of exposure (eg, dead poultry). Clinicians should be aware of meningoencephalitis associated with A(H5N1) infection in the absence of respiratory symptoms.

## Supplementary Material

ofag283_Supplementary_Data

## Supplementary Data

Supplementary materials are available at *Open Forum Infectious Diseases* online. Consisting of data provided by the authors to benefit the reader, the posted materials are not copyedited and are the sole responsibility of the authors, so questions or comments should be addressed to the corresponding author.

## APPENDIX

**Members of the SEACOVARIANTS Consortium**

Nguyen To Anh^1^, Nguyen Thi Thu Hong^1^, Truong Hoang Chau Truc^1^, Nguyen Thi Han Ny^1^, Do Duong Kim Han^1^, Le Kim Thanh^1^, Lam Anh Nguyet^1^, Cao Thu Thuy^1^, Le Nguyen Truc Nhu^1^, Tran Tan Thanh^1^, Lam Minh Yen^1^, Vu Thi Ty Hang^1^, Pham Ngoc Long^1^, Pham Tieu Kieu^1^, Vo Tan Hoang^1^, Nguyen Thi Thao^1^, Mary Chambers^1^, Vu Duy Thanh^1^, Pham Ngoc Long^1^, H. Rogier van Doorn^2,3^, Trinh Son Tung^2^, C. Louise Thwaites^1,3^, Guy Thwaites^1,3^, Lin-Fa Wang^4^, Beng Lee Lim^4^, Raph L. Hamers^3,5^, Anuraj Shankar^3,5^, Suwatrti Suwarti^5^, Yanie Tayipto^5^, Eva Simarmata^5^, Ragil Dien^5^, Juthathip Mongkolsapaya^6^, Wanwisa Dejnirattisai^7^, Warangkana Chantima^8^, Narisara Chantratita^9^, Prapassorn Poolchanuan^9^, Vichapon Tiacharoen^9^, Adul Dulsuk^9^, Sophon Iamsirithaworn^10^, Nick Day^11^, Phaik Yeong Cheah^11^, Tassawan Poomchaichote^11^, Kanpong Boonthaworn^11^, Gavin Screaton^6^, Aiete Dijokaite-Guraliuc^6^, Raksha Das^6^, Chang Liu^6^, Piyada Supasa^6^, Muneeswaran Selvaraj^6^, Susanna J Dunachie^12^, Paul Klenerman^12^, E. Yvonne Jones^12^, David I. Stuart^12^, Barbara Kronsteiner-Dobramysl^12^, Martha Zewdie^12^, Priyanka Abraham^12^, Jennifer Hill^12^, Nghiem My Ngoc^13^, Alba Grifoni^14^, Alessandro Sette^14^, Wee Chee Yap^15^, Chee Wah Tan^15^, and Le Van Tan^1^

^1^Oxford University Clinical Research Unit, Ho Chi Minh City, Vietnam, ^2^Oxford University Clinical Research Unit, Ha Noi, Vietnam, ^3^Centre for Tropical Medicine and Global Health, Nuffield Department of Medicine, University of Oxford, Oxford, UK, ^4^Programme for Emerging Infectious Diseases, Duke-NUS Medical School, Singapore, ^5^Oxford University Clinical Research Unit Indonesia, Faculty of Medicine Universitas Indonesia, Jakarta, Indonesia, ^6^Wellcome Centre for Human Genetics, Nuffield Department of Medicine, University of Oxford, Oxford, UK, ^7^Division of Emerging Infectious Disease, Research Department, Faculty of Medicine Siriraj Hospital, Mahidol University, Bangkok, Thailand, ^8^Siriraj Center of Research Excellence in Dengue and Emerging Pathogens, Faculty of Medicine Siriraj Hospital, Mahidol University, Bangkok, Thailand, ^9^Department of Microbiology and Immunology, Faculty of Tropical Medicine, Mahidol University, Bangkok, Thailand, ^10^Department of Disease Control, Ministry of Public Health, Thailand, ^11^Mahidol Oxford Tropical Medicine Research Unit, Bangkok, Thailand, ^12^Nuffield Department of Medicine, University of Oxford, Oxford, UK, ^13^Hospital for Tropical Diseases, Ho Chi Minh City, Vietnam, ^14^La Jolla Institute for Immunology, La Jolla, California, USA, ^15^Yong Loo Lin School of Medicine, National University of Singapore

**Members of the H5N1 Consortium**

Isabella Oyier^1^, Alex Sigal^2^, Marvin Hsiao^3^, Muki Shey^3^, Jennifer Cornick^4^, Ndaru Jambo^4^, Elizabeth Batty^5^, Narisara Chantratita^6^, Juthatip Mongkolsapaya^5^, Raph Hamers^7^, Tao Dong^8^, Susanna Dunachie^9^, Alba Grifoni^10^, Alessandro Sette^10^, Wanwisa Dejnirattisai^11^, Sebastian Maurer-Stroh^12^, Warangkana Chantima^11^, Robert J. Wilkinson^3^, Sandy Mak^12^, Raymond Moseki^3^, Guihai Liu Charles Nyaigoti^1^, Suwarti, Chee Wah Tan^13^, Gavin Screaton^14^, Truong Hoang Chau Truc^15^, Tran Tan Thanh^15^, Nguyen To Anh^15^, and Le Van Tan^15^

^1^KEMRI–Wellcome Trust Research Programme, ^2^Africa Health Research Institute, South Africa, ^3^Centre for Infectious Diseases Research in Africa, University of Cape Town, South Africa, ^4^Malawi Liverpool Wellcome Research Programme, Malawi, ^5^Mahidol Oxford Tropical Medicine Research Unit, Bangkok, Thailand, ^6^Department of Microbiology and Immunology, Faculty of Tropical Medicine, Mahidol University, Bangkok, Thailand, ^7^Oxford University Clinical Research Unit, Faculty of Medicine Universitas Indonesia, Jakarta, Indonesia, ^8^Chinese Academy of Medical Sciences Oxford Institute, Nuffield Department of Medicine, University of Oxford, Oxford, UK, ^9^Nuffield Department of Medicine, University of Oxford, Oxford, UK, ^10^La Jolla Institute for Immunology, La Jolla, California, USA, ^11^Division of Emerging Infectious Disease, Research Department, Faculty of Medicine Siriraj Hospital, Mahidol University, Bangkok, Thailand, ^12^A*STAR Bioinformatics Institute (BII), Agency for Science, Technology and Research, Singapore, ^13^Infectious Diseases Translational Research Programme, Yong Loo Lin School of Medicine, National University of Singapore, Singapore, ^14^Wellcome Centre for Human Genetics, Nuffield Department of Medicine, University of Oxford, Oxford, UK, ^15^Oxford University Clinical Research Unit, Ho Chi Minh City, Vietnam.
