# Supplementary material for: Central Nervous System Involvement by Novel Clade 2.3.2.1e H5N1 Avian Influenza Virus in a Pediatric Patient
Source: Open Forum Infect Dis. 2026 May 7;13(5):ofag283. doi: 10.1093/ofid/ofag283 (PMC13197838; doi:10.1093/ofid/ofag283)
Supplement: ofag283_Supplementary_Data [file ofag283_supplementary_data.docx]

**Appendix**

**Central Nervous System Involvement by Novel Clade 2.3.2.1e H5N1 Avian Influenza Virus in a Paediatric Patient**

**MATERIALS AND METHODS**

**Clinical study**

The present study forms part of an ongoing observational study of emerging and re-emerging infectious diseases that has been conducted in collaboration between the Oxford University Clinical Research Unit, the Hospital for Tropical Diseases and Children’s Hospital 1, in Ho Chi Minh City, Vietnam. The patients and their household contacts had clinical samples (including respiratory and blood samples) collected at enrollment alongside clinical and demographic data. At discharge a blood sample was also collected from the study participants for assessment of immune response. Additionally, leftovers of clinical samples after routine diagnosis were also stored for the study purpose.

**Clinical management**

The patient was managed and isolated according to the local practice and national guidelines. Accordingly for the treatments mentioned, standard doses were applied unless otherwise stated. Accordingly, 150 mg oseltamivir was given twice daily for 16 days in the contex of low CSF penetration[1].

**Chest X-ray and MRI assessments**

Chest X-ray and brain MRI assessments were carried out by radiologists and senior clinicians at CH1 who were also looking after the patient. Conclusions were drawn based on the consensuses agreed among the assessors.

**Ethics**

The Institutional Review Boards of the Hospital for Tropical Diseases and Children’s Hospital 1 and the Oxford Tropical Research Ethics Committee approved the study, with respective approval codes of CS/BND/20/01, CS/N1/25/34 and 1023‐13. For the participation of the patient in our study, a written informed consent was obtained from a parent, and as per our local practice a written assent form was also obtained from the patient. Written informed consent forms were obtained from both parents who were close contacts of the patient before they participated in the study.

**List of pathogens covered by multiplex real time RT-PCR platform (Nam Khoa Diagnostics, Ho Chi Minh City, Vietnam) conducted as part of routine diagnostic workup**

Viruses

Herpes simplex virus type 1&2, Lymphocytic choriomeningitisNP2&GP, Cytomegalovirus, Varicella-Zoster Virus, Epstein-Barr Virus, Human herpesvirus 6, Enterovirus A71, Echovirus, Coxackievirus A&B, Saffold virus, Human parechovirus, Japenese encephalitis virus, influenza A virus, Measles virus, Rubella virus, Rabies virus, Zika virus, Mumps virus, Parvovirus B19, John Cunningham virus, BK virus, Adenovrus and Dengue virus

Bacteria

*Neiseria meningitidis, Streptococcus pneumoniae, Streptococcus suis, Streptococcus pyogenes, Streptococcus agalactiae, Haemophilus influenzae type B, Salmonella sp., Elizabethkingia meningoseptica, Listeria monocytogenes, Escherichia coli, Escherichia coli K1, Brucella, Treponema pallidum, Leptospira interrogans, Rickettsia prowazekii, Orientia tsutsugamushi,* and *Actinomyces israelii*

Mycobacteria

*Mycobacterium tuberculosis, and Non- Mycobacterium tuberculosis*

Parasites

*Acanthamoeba, Naegleria fowleri, Balamuthia mandrillaris, Entamoeba histolytica, Baylisascaris procyonis, Toxoplasma gondii, Angiostrongylus cantonensis, Strongyloides stercoralis, Taenia solium* and *Gnathostoma spinigerum*

Fungi

*Cryptococcus neoformans, Pneumocystis carinii, Penicillium marneffei, Coccidioides immitis/posadasii, Scotoplanes globose, Sporothrix schenckii/brasiliensis, Mucormycosis* and *Candida glabrata*

Multiplex real-time RT-PCR testing for the abovementioned pathogens was carried out as part of routine diagnosis, and accordingly the admission CSF was submitted to the laboratories of Nam Khoa BioTek and Vietnam Institute of Research and Development Clinical Microbiology for testing[2]. After routine diagnosis finding, follow-up samples were tested using US CDC IAV and A/H5 and commercial assays as detailed below.

**Nucleic acid isolation for detection of influenza A virus and its subtypes (H3, pdmH1 and H5)**

Viral RNA was isolated from 140uL of clinical samples using QIAamp Viral RNA Mini kit (QIAgen GmbH, Hilden, Germany), following the manufacture’s instruction. Isolated viral RNA was then eluted in 50uL of the elution buffer provided with the kit and was used for PCR diagnostics and whole genome sequencing where appropriate.

**Multiplex real time RT-PCR conducted after routine diagnostic findings**

Detection of influenza A virus and its subtypes (H3, pdmH1 and H5) was carried out using the US CDC recommended assays. Primer and probe sequences are listed in Table S3. For detection of common causes of central nervous system infections in follow up CSF samples collected after routine diagnostics findings, we used three multiplex PCR panels (Seegene, Seoul, South Korea), including Allplex^TM^ Meningitis-V1 Assay (Cat. No. MG9700X, MG10209Z); Allplex^TM^ Meningitis-V2 Assay (Cat. No. MG9500X, MG10210Z), and Allplex^TM^ Meningitis-B Assay (Cat. No. MG9600X, MG10211Z) were used. These three panels cover 12 viruses and 6 bacteria that are known to cause central nervous system infections, including herpes simplex virus 1&2, varicella zoster virus, Epstein-Barr virus, cytomegalovirus, human herpes virus 6&7, adenovirus, human parechovirus, enteroviruses, mumps virus, parvovirus B19, *Neisseria meningitidis*, *Listeria monocytogenes*, *Haemophilus influenzae*, *Streptococcus agalactiae*, *Streptococcus pneumoniae*, *Escherichia coli K1*.

**Measurement of binding IgG against HA1 subunit**

To measure we used microsphere immunoassay[3-5]. In brief, influenza A virus hemagglutinin HA1 protein of H5N1 (A/Cambodia/i0125001G/2024) was produced in-house using HEK293T cells and purified with Ni-Sepharose (Cytiva). The HA1 proteins were enzymatically biotinylated and coated onto Avidin-conjugated Magplex Microspheres (Luminex). HA1-specific antibodies were detected by pre-incubating 1:400-diluted patient heat-inactivated CSF and plasma samples at 37°C for 1 hour with agitation, followed by three washes. Anti-human IgG-PE was then added at a final dilution of 1:500 to detect IgG antibodies. The signal was acquired using a MAGPIX instrument, and the results were expressed as Net Mean Fluorescence Intensity (Net MFI). A value of > or =1000 Net MFI was selected as the cut-off value for positivity, selected based on the background signals obtained during the validation step (data not shown).

**Control CSF and plasma samples**

For negative controls used as part of the Luminex experiments measuring antibodies against HA1 subunit, we use 5 CSF and 5 plasma samples derived from a previously described CNS infection diagnostics study[6] and a study of COVID-19 vaccine evaluation in Vietnamese healthcare workers[7]. For positive control, we used a convalescent plasma sample collected from a patient with PCR confirmed A(H5N1) virus infection.

**Whole genome sequencing**

To whole genome sequence the virus directly from the PCR positive CSF samples, we first amplify all 8 segments of the virus from leftover RNA isolated from the CSF collected on day 4 of hospitalization using Tuni 12 and Tuni 13 primers[8]. The amplified products were then cleaned up and used as input for whole-genome sequencing. For this step, Illumina COVIDSeq reagents (Illumina) were used to generate DNA library, following manufacturer’s instructions. The concentration and size of the constructed library were accessed using Qubit dsDNA HS kit (Invitrogen) and Agilent High Sensitivity D1000 kit (Aligent Technologies), respectively. Finally, the prepared library was sequenced using MiSeq Micro kit V2 (300 cycles) in a Miseq platform.

**Sequence analysis**

The obtained reads coming out of the Illumina MiSeq were subjected whole genome sequence assembly using Iterative Refinement Meta-Assembler (IRMA)[9]. The obtained consensuses were further manually edited to very any ambiguous positions (where appropriate).

Clade assignments of the HA sequence obtained from the patient were done using a combination of NextClade (<https://clades.nextstrain.org/>).

Phylogenetic analyses of individual segments were carried out using IQ-Tree[10], using non-identical sequences selected from top 100 best hits of the corresponding segments informed by EpiFlu and representatives of clade 2.3.4.4b sequences (accessed on April 2025). Screening for amino acid substitutions with biological significance potential was carried using Flusurver (<https://flusurver.bii.a-star.edu.sg/>) and FluMut[11]. For Flusurver outputs, only substitutions compared to closest reference sequences and marked as warn-level 2 (significant) or 3 (most significant) were considered, and those with unknown or neutral effect were removed from the final list. A substitution is marked as warn level 2 when its equivalent site is known to result in antigenic shifts or causes mild drug resistance or if it occurs at a site known to involved in drug-binding or alters host-cell specificity. A substitution is marked as warn-level 3 if it is known to alter the virulence of the virus, cause strong drug resistance or reverses the effects of the premature STOP codon in the PB1-F2 gene of pandemic H1N1. The outputs generated by both tools are compiled into a single report, and the observed mutations were further assessed for their prevalence in sequences submitted to GISAID. For this analysis we focused on non-duplicated sequences of clade 2.3.2.1c/e viruses with all 8 segments submitted GISAID, which already contained sequences of the present study.

**GISAID submission**

The obtained whole genome sequence of the viral strain was submitted to GISAID under the ID number: EPI_ISL_19850663

**RESULTS**

**The patient**

The patient is a previously healthy 8-year-old boy. He came from Tay Ninh province in southern Vietnam. Geographically, Tay Ninh province borders Cambodia where recent H5N1 patients have been reported over the last two years. In the past, the patient underwent a successful heart surgery at 2 months of age because of ventricular septal defect. However, currently, he is not on any cardiovascular drugs. A cardiac ultrasound performed on admission showed the contraction of the heart was effective, with no shunts and no murmur observed.

**Table S1:** Laboratory findings of blood samples collected on admission and during hospitalization

|  | Normal range | Blood 1 (admission to local hospital), illness day 1 | Blood 2 (admission to CH1),  illness day 3 | Blood 3,  illness day 5 | Blood 4,  illness day 8 | Blood 5,  illness day 10 | Blood 6,  illness day 11 | Blood 7,  illness day 14 |
| --- | --- | --- | --- | --- | --- | --- | --- | --- |
| Leukocyte count (cells/μL) | 4,500-14,500 | 23,700 | 10,860 | 5150 | 10,140 | 15,680 | Not done | 11,810 |
| Hemoglobin (g/dL) | 11.5-14.0 | 11.8 | 11.5 | 11.5 | 10.6 | 9.7 | Not done | 10.9 |
| Platelet count (cells/μL) | 150,000-400,000 | 456,000 | 318,000 | 399,000 | 399,00 | 407,000 | Not done | 878,000 |
| C reactive protein (mg/dL) | =<0.9 | 0.36 | 97.24 | Not done | Not done | 100.74 | 37.90 | 6.93 |
| Procalcitonin (ng/mL) | =<0.05 | Not done | Not done | 32.550 | 7.963 | Not done | 2.165 | 0.13 |
| Aspartate aminotransferases (U/liter) | <50 | 21 | 22.2 | Not done | Not done | 22.29 | Not done | 21.28 |
| Alanine aminotransferase (U/liter) | <55 | 11.8 | 11.6 | Not done | Not done | 19.57 | Not done | 23.22 |
| Creatinine (mg/dL ) | 30-47 | Not done | 48.1 | Not done | Not done | 36.03 | Not done | 36.3 |

**Table S2:** Primer and probe sequences

| **Target** | **Oligo name** | **Oligonnucleotide sequence (5’>3’)** |
| --- | --- | --- |
| Influenza A viruses | InfA For1 | CAA GAC CAA TCY TGT CAC CTC TGA C |
|  | InfA For2 | CAA GAC CAA TYC TGT CAC CTY TGA C |
|  | InfA Rev1 | GCA TTY TGG ACA AAV CGT CTA CG |
|  | InfA Rev2 | GCA TTT TGG ATA AAG CGT CTA CG |
|  | InfA Pro | FAM-TGC AGT CCT CGC TCA CTG GGC ACG-BHQ1 |
| Influenza B viruses | InfB- For | TCC TCA AYT CAC TCT TCG AGC G |
|  | InfB- Rev | CGG TGC TCT TGA CCA AAT TGG |
|  | InfB Pro | FAM-CCA ATT CGA GCA GCT GAA ACT GCG GTG-BHQ1 |
| A/pdm H1 | pdmH1-F | GTG CTA TAA ACA CCA GYC TCC CAT T |
|  | pdmH1-R | AGA YGG GAC ATT CCT CAA TCC TG |
|  | pdmH1-P | FAM-TGG CCA GYC “T”CA ATT TTG TGC TTT TTA CAT A-BHQ1 |
| A/H3 | H3 For | AAG CAT TCC YAA TGA CAA ACC |
|  | H3 Rev | ATT GCR CCR AAT ATG CCT CTA GT |
|  | H3-Pro | FAM-CAG GAT CAC A“T”A TGG GSC CTG TCC CAG-BHQ1 |
| A/H5 | H5a For 1 | TGG AAA GTG TRA GAA ACG GRA CRT A |
|  | H5a For 2 | TGG AAA GTA TAA GRA ACG GAA CRT A |
|  | H5a For 3 | TGG ARA GYG TAA GAA ATG GGA CGT A |
|  | H5a Rev 1 | CTA GGG ARC TCG CCA CTG TWG A |
|  | H5a Rev 2 | CTA GDG AAC TCG CAR CTG TTG A |
|  | H5a Pro 1 | FAM-TGA CTA CCC GCA G"T"A TTC AGA AGA AKC AAG AYT AA-BHQ1 |
|  | H5a Pro 2 | FAM-CAA CTA TCC GCA G"T"A TTC AGA AGA AGC AAG ATT AA-BHQ1 |
|  | H5a Pro 3 | FAM-TGA CTA CCC "T"AA GTA TTC AGA AGA AGC AAK ATT AA-BHQ1 |
|  | H5b For1 | GGA ATG YCC CAA ATA TGT GAA ATC AA |
|  | H5b For2 | GGA RTG CCC CAA ATA CGT GAA RTC AA |
|  | H5b-Rev | CCR CTC CCC TGC TCR TTR CT |
|  | H5b- Pro | FAM-TAC CCA “T”AC CAA CCA TCT ACC ATY CCC TGC CAT-BHQ1 |

**Table S3**: Duration of antivirals and antibiotics used

| Antibiotics | Durations of administration in days |
| --- | --- |
| Ceftriaxone | 1 |
| Meropenem | 16 |
| Linezolid | 12 |
| Levofloxacin | 12 |
| Vancomycin | 3 |
| Acyclovir | 5 |
| Oseltamivir | 13 |

**Table S4:** Results of PCR analysis of serial samples of the patient

| **Sample types** | **Sampling time: illness day: 3** | | **Sampling time: illness day: 6** | | | **Sampling time: illness day: 8** | | | **Sampling time: illness day: 11** | | | **Sampling time: illness day: 12** | | | **Sampling time: illness day: 18** | | |
| --- | --- | --- | --- | --- | --- | --- | --- | --- | --- | --- | --- | --- | --- | --- | --- | --- | --- |
|  | **Sample type** | **Flu A PCR (Ct)*** | **Sample type** | **Flu A PCR (Ct)** | **H5 PCR (Ct)** | **Sample type** | **Flu A PCR (Ct)** | **H5 PCR (Ct)** | **Sample type** | **Flu A PCR (Ct)** | **H5 PCR (Ct)** | **Sample type** | **Flu A PCR (Ct)** | **H5 PCR (Ct)** | **Sample type** | **Flu A PCR (Ct)** | **H5 PCR (Ct)** |
| CSF | Y | Positive (19) | Y | Positive (26) | Positive (34) | Y | Positive (31) | Negative | NA | NA | NA | Y | Positive (34) | Negative | Y | Negative | ND |
| ETA | N | NA | Y | Negative | Negative | NA | Negative | ND | NA | NA | NA | NA | NA | NA | N | NA | NA |
| Plasma | N | NA | N | NA | NA | Y | Negative | ND | Y | Negative | ND | NA | NA | NA | N | NA | NA |
| Serum | N | NA | N | NA | NA | Y | Negative | ND | NA | NA | NA | NA | NA | NA | N | NA | NA |
| Urine | N | NA | N | NA | NA | Y | Negative | ND | Y | Negative | ND | NA | NA | NA | N | NA | NA |
| Throat swab | N | NA | N | NA | NA | Y | Negative | ND | Y | Negative | ND | NA | NA | NA | N | NA | NA |
| Rectal swab | N | NA | N | NA | NA | Y | Negative | ND | Y | Negative | ND | NA | NA | NA | N | NA | NA |

**Notes to Table S4:** CSF: cerebrospinal fluid, ETA: Endotracheal aspirate NA: non-applicable, N: no, Y: yes, ND: not done. IAV PCR is more sensitive than A/H5 therefore, as per our workflow, A/H5 PCR is carried out only if IAV PCR is positive. *as part of the routine diagnostic workup and A/H5 PCR was not done. Obtaining serial CSF samples were based on the basis of clinical progression (the first three lumbar punctures) and local public health measures (the last two), requiring that the patients tested negative for the virus before discharge.

PCR testing for common cause of CNS infection using the Seegene assays on follow-up CSF samples only return EBV with viral load at the borderline of the PCR detection limit; Ct values: 39 for CSF collected on 16 April 2025, 34 for CSF sample collected on 18 April 2025, and 40 for CSF sample collected on 22 April 2025 (Supplementary Materials). CSF sample collected on 28 April 2025 was negative for EBV. These findings likely reflect the circulation of white blood cells carrying EBV in the CSF rather a pathological association with the observed clinical presentation.

**Table S5:** List of best hits of the corresponding segments generated by EpiFlu tool

| **Segment** | **Closest sequences** | **Isolate ID** | **Segment ID** | **Identity %** |
| --- | --- | --- | --- | --- |
| 1 (PB2) | A/Cambodia/SVH240441/2024 | EPI_ISL_19312044 | EPI3513544 | 99.87 |
| 2 (PB1) | A/Cambodia/SVH240441/2024 | EPI_ISL_19312044 | EPI3513543 | 99.87 |
| 3 (PA) | A/Cambodia/SVH240441/2024 | EPI_ISL_19312044 | EPI3513545 | 99.67 |
| 4 (HA) | A/Cambodia/SVH240441/2024 | EPI_ISL_19312044 | EPI3489221 | 99.36 |
| 5 (NP) | A/Cambodia/SVH240441/2024 | EPI_ISL_19312044 | EPI3489217 | 99.87 |
| 6 (NA) | A/Cambodia/SVH240441/2024 | EPI_ISL_19312044 | EPI3489220 | 99.48 |
| 7 (MP) | A/Cambodia/SVH240441/2024 | EPI_ISL_19312044 | EPI3489219 | 99.80 |
| 8 (NS) | A/Cambodia/SVH240441/2024 | EPI_ISL_19312044 | EPI3489218 | 99.64 |

**Table S6:** List of mutations with biological significance potential detected by Flusurver and FLuMut

| **Mutation** | **Prevalence of mutation* (n)** | **Prevalence of mutation (%) (n/1114)** | **Annotation method** | **Effect** | **Subtype** | **Literature** |
| --- | --- | --- | --- | --- | --- | --- |
| PB2:E627K | 37 | 3.32 | FluMut, FluSurver | Contributes to airborne pathogenicity in ferrets;  Involved in host specificity shift; Virulence;  Viral oligomerization interfaces,  Binding small ligand(s) | H5N1 | Bogs J. et al., 2011; Bortz E. et al., 2011; Chen H. et al., 2007;  Fornek J. et al., 2009; Hatta H. et al., 2001;  Hatta M. et al., 2007; Herfst S. et al., 2012; Kim J. et al., 2010; Le Q. et al., 2005; Long J. et al., 2013; Manzoor R. et al., 2009; Mase M. et al., 2006; Richard M. et al., 2017;  Shinya K. et al., 2004; Suttie A. et al., 2019 |
| PB2:L89V, PB2:G309D | 1057 | 94.88 | FluMut | Increased polymerase activity in mammalian cells | H5N1 | Li J. et al., 2009; Suttie A. et al., 2019 |
| PB2:A676T | 775 | 69.57 | FluSurver | Contributed to the virulence and adaptation in mice in combination with other PB2 mutations | H5N1 | Li J. et al., 2009 |
| PB2:S715N | 1070 | 96.05 | FluMut, FluSurver | Decreased virulence in mice | H5N1 | Sun H. et al., 2015; Suttie A. et al., 2019 |
| PB2:V598T | 930 | 83.48 | FluMut | Increased polymerase activity in mammalian cells | H7N9 | Hu M. et al., 2017b; Suttie A. et al., 2019 |
| PB1:D3V | 1012 | 90.84 | FluMut | Increased polymerase activity in avian cells | H5N1 | Elgendy E. et al., 2017; Suttie A. et al., 2019 |
| PB1:D622G | 1085 | 97.40 | FluMut | Increased polymerase activity in mammalian cells | H5N1 | Feng X. et al., 2016; Suttie A. et al., 2019 |
| PB1:H115Q, PB2:E627K | 37 | 3.32 | FluMut | Transmitted via aerosol among guinea pigs | H7N9 | Zhang C. et al., 2021 |
| PB1-F2:N66S | 165 | 14.81 | FluMut | Enhanced antiviral response in mice | H5N1 | Conenello G. et al., 2007; Schmolke M. et al., 2011;  Suttie A. et al., 2019 |
| PA:K142N | 54 | 4.85 | FluMut, FluSurver | Increased virulence in mice; involved in binding small ligands | H5N1 | Kim J. et al., 2010; Suttie A. et al., 2019 |
| PA:N383D | 1093 | 98.11 | FluMut | Increased polymerase activity in avian cells | H5N1 | Song J. et al., 2011; Song J. et al., 2015; Suttie A. et al., 2019 |
| PA:N409S | 1053 | 94.52 | FluMut | Increased polymerase activity in mammalian cells | H7N9 | Suttie A. et al., 2019; Yamayoshi S. et al., 2014 |
| PA:P190S | 1079 | 96.86 | FluMut | Decreased virulence in mice | H7N3 | DesRochers B. et al., 2016; Suttie A. et al., 2019 |
| PA:S37A | 1044 | 93.72 | FluMut | Increased polymerase activity in mammalian cells | H7N9 | Suttie A. et al., 2019; Yamayoshi S. et al., 2014 |
| HA:D94N | 1047 | 93.99 | FluMut, FluSurver | Increased pseudovirus binding to α2,6 | H5N1 | Su Y. et al., 2008; Suttie A. et al., 2019 |
| HA:K64E | 1113 | 99.91 | FluMut | Decreased HA stability | H7N9 | Sun X. et al., 2019; Suttie A. et al., 2019 |
| HA:S107R, HA:T108I | 1087 | 97.58 | FluMut | Increased pH of fusion | H5N1 | Suttie A. et al., 2019; Wessels U. et al., 2018 |
| HA:S123P, HA:R167K | 1** | 0.09 | FluMut, FluSurver | Increased virus binding to α2,6 | H5N1 | Suttie A. et al., 2019; Yamada S. et al., 2006 |
| HA:S133A | 1101 | 98.83 | FluMut | Increased pseudovirus binding to α2,6 | H5N1 | Suttie A. et al., 2019; Yang Z. et al., 2007 |
| HA:S154N | 513 | 46.05 | FluMut | Increased virus binding to α2,6 | H5N1 | Suttie A. et al., 2019; Wang W. et al., 2010 |
| HA:S155N | 997 | 89.50 | FluMut | Increased virus binding to α2,6 | H5N1 | Suttie A. et al., 2019; Wang W. et al., 2010 |
| HA:S155N, HA:T156A | 966 | 86.71 | FluMut | Increased virus binding to α2,6 | H5N1 | Suttie A. et al., 2019; Wang F. et al., 2015;  Wang W. et al., 2010 |
| HA:T134A | 1098 | 98.56 | FluMut | Increased viral replication in mice lungs | H9N2 | Zhang J. et al., 2023 |
| HA:T156A | 999 | 89.68 | FluMut | Increased transmission in guinea pigs | H5N1 | Gao Y. et al., 2009; Suttie A. et al., 2019; Wang W. et al., 2010 |
| HA:V182N | 1089 | 97.76 | FluMut | Decreased virus binding to α2,3 | H13N6 | Lu X. et al., 2013; Suttie A. et al., 2019 |
| NP:A184K | 1089 | 97.76 | FluMut | Enhanced interferon response | H5N1 | Suttie A. et al., 2019; Wasilenko J. et al., 2009 |
| NP:M105V | 1066 | 95.69 | FluMut | Increased virulence in chickens | H5N1 | Suttie A. et al., 2019; Tada T. et al., 2011; Tada T. et al., 2011b |
| NA:Y155H | 1053 | 94.52 | FluMut | Highly reduced inhibition to Oseltamivir | H1N1 | Monto A. et al., 2006 |
| M1:I43M | 1109 | 99.55 | FluMut | Increased virulence in chickens | H5N1 | Nao N. et al., 2015; Suttie A. et al., 2019 |
| M1:N30D | 1096 | 98.38 | FluMut | Increased virulence in mice | H5N1 | Fan S. et al., 2009; Suttie A. et al., 2019 |
| M1:T215A | 1112 | 99.82 | FluMut | Increased virulence in mice | H5N1 | Fan S. et al., 2009; Suttie A. et al., 2019 |
| NS1:C138F | 1086 | 97.49 | FluMut | Decreased interferon response | H5N1 | Li J. et al., 2018; Suttie A. et al., 2019 |
| NS1:C138F, NS1:K55E, NS1:K66E | 940 | 84.38 | FluMut | Decreased interferon response | H5N1 | Li J. et al., 2018; Suttie A. et al., 2019 |
| NS1:I106M | 1107 | 99.37 | FluMut | Increased viral replication in mammalian cells | H1N1 with all internal genes from H7N9 | Ayllon J. et al., 2014; Suttie A. et al., 2019 |
| NS1:L103F, NS1:I106M | 1060 | 95.15 | FluMut | Increased virulence in mice | H5N1 | Kuo R. et al., 2009; Spesock A. et al., 2011;  Suttie A. et al., 2019 |
| NS1:N205S, NS2:T48A | 436 | 39.14 | FluMut | Decreased antiviral response in ferrets | H5N1 | Imai H. et al., 2010; Suttie A. et al., 2019 |
| NS1:P42S | 1099 | 98.65 | FluMut | Decreased antiviral response in mice | H5N1 | Jiao P. et al., 2008; Suttie A. et al., 2019 |
| NS1:V149A | 1113 | 99.91 | FluMut | Decreased interferon response in chickens | H5N1 | Li Z. et al., 2006; Suttie A. et al., 2019 |

**Note to Table S6:** Listed mutations are those with biological significance potential that have been shown for the specified IAV subtype, which might not be applicable for other subtypes. For examples, NA Y155H is associated with highly reduced inhibition by neuraminidase inhibitors in the seasonal IAV A/H1N1 circulating before the 2009 pandemic. However, this substitution did not confer a phenotype of reduced susceptibility to neuraminidase inhibitors in influenza A(H1N1)pdm09 viruses[12]

* Total 1114 sequences of clade 2.3.2.1c/e were analyzed, **sequence of the present study


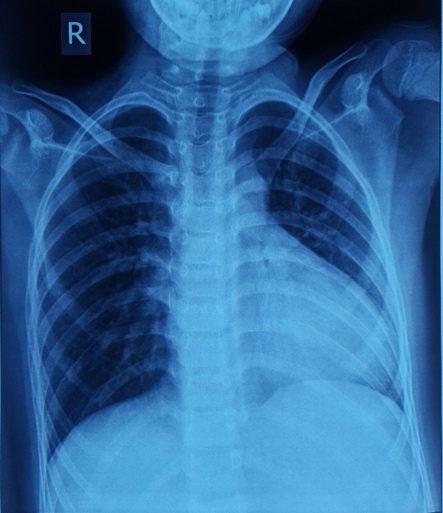


**Figure S1:** Chest radiograph showing consolidation in the left lower lobe of the lung. Subsequent chest radiographs taken on day 2 and 5 of hospitalisation revealed similar finding of the chest radiograph obtained on admission (data not shown).


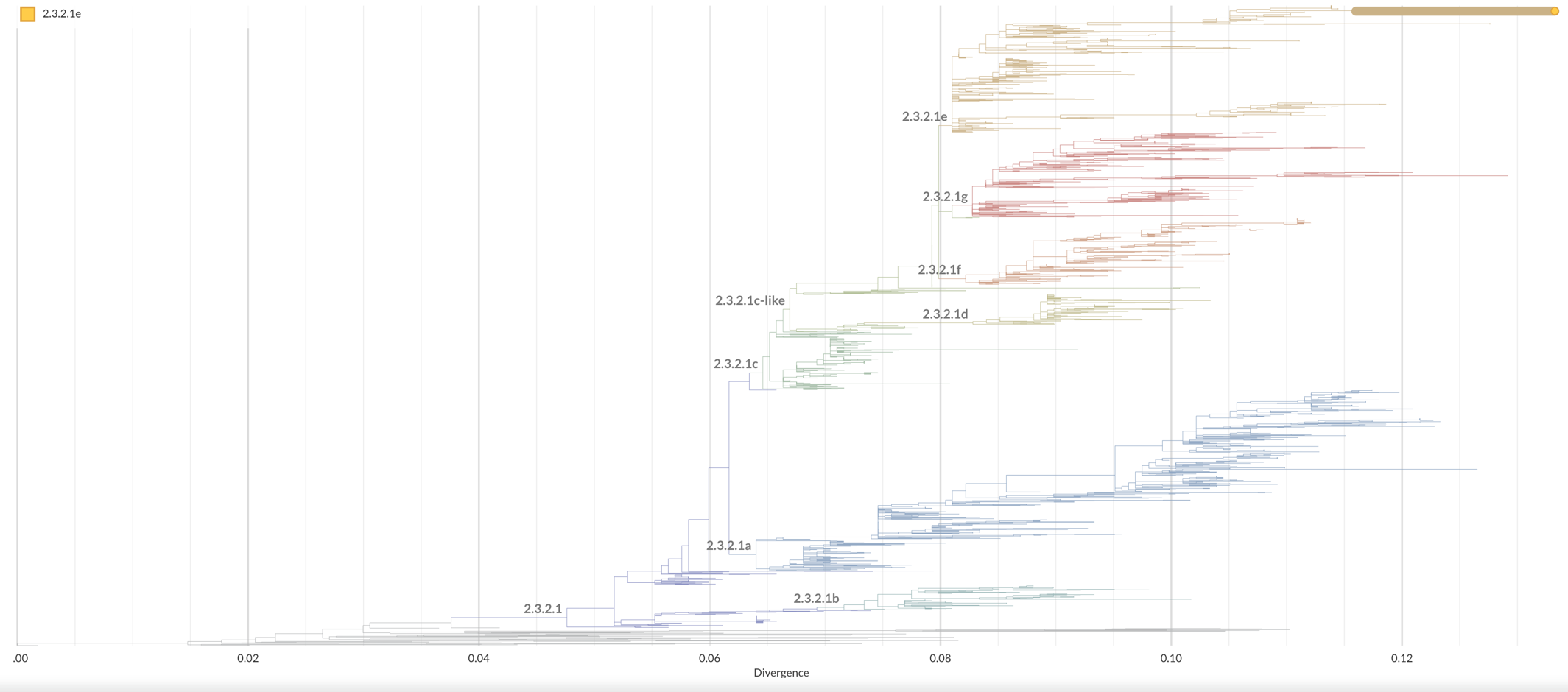


**Figure S2**: Screenshot showing the relative placement of the HA sequence of the present study (indicated by arrow) in the global tree context, using NextClade tool.

**B**


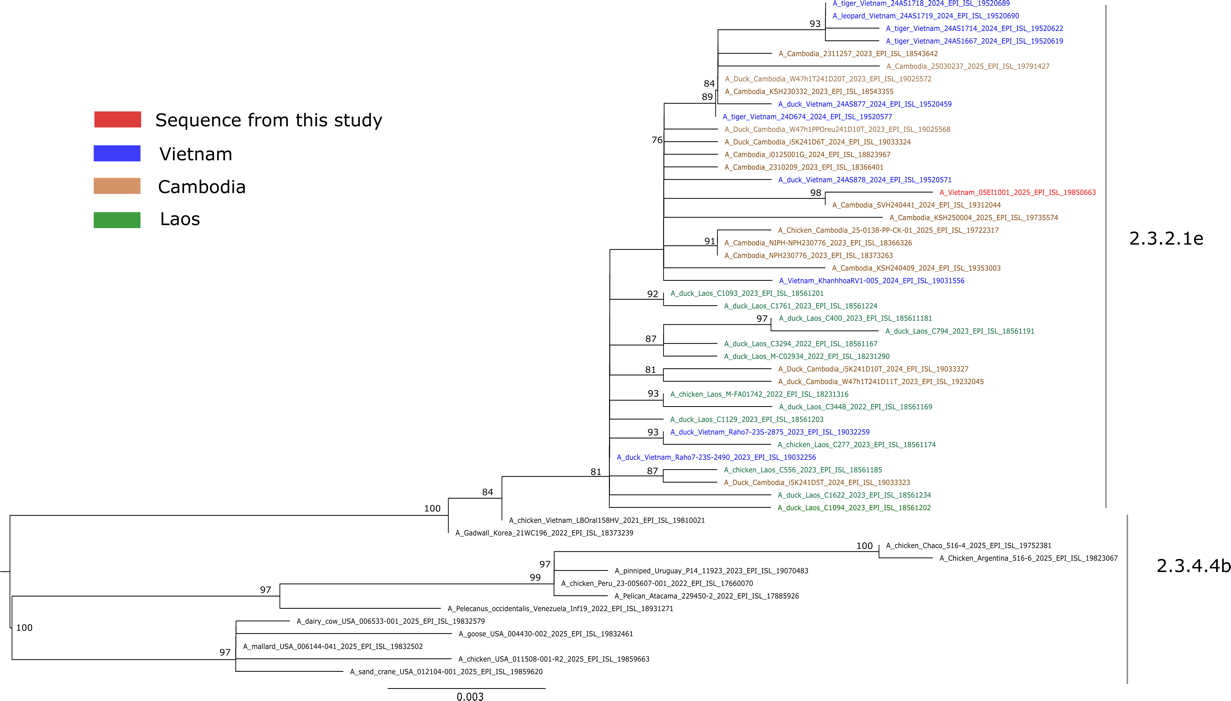


**A**


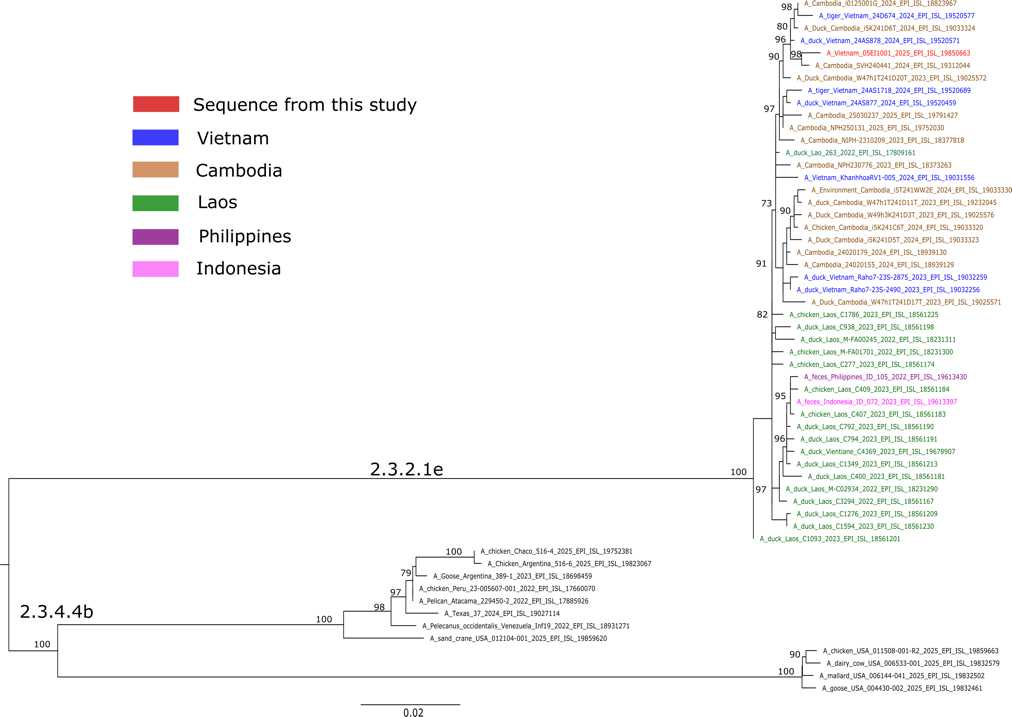

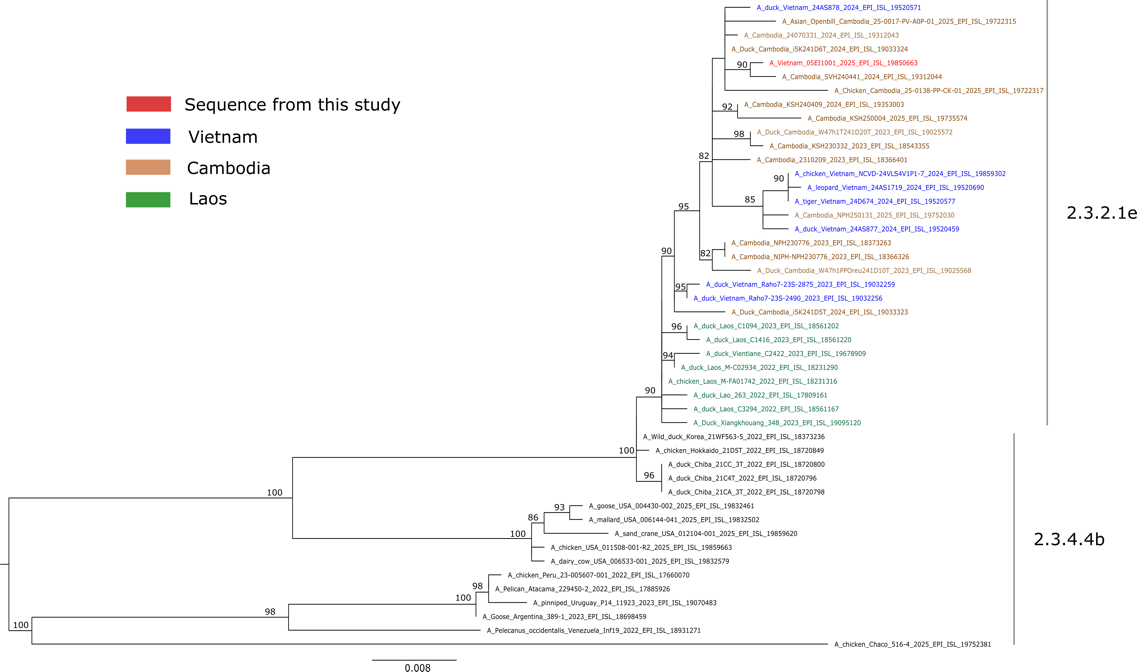


**D**

**C**


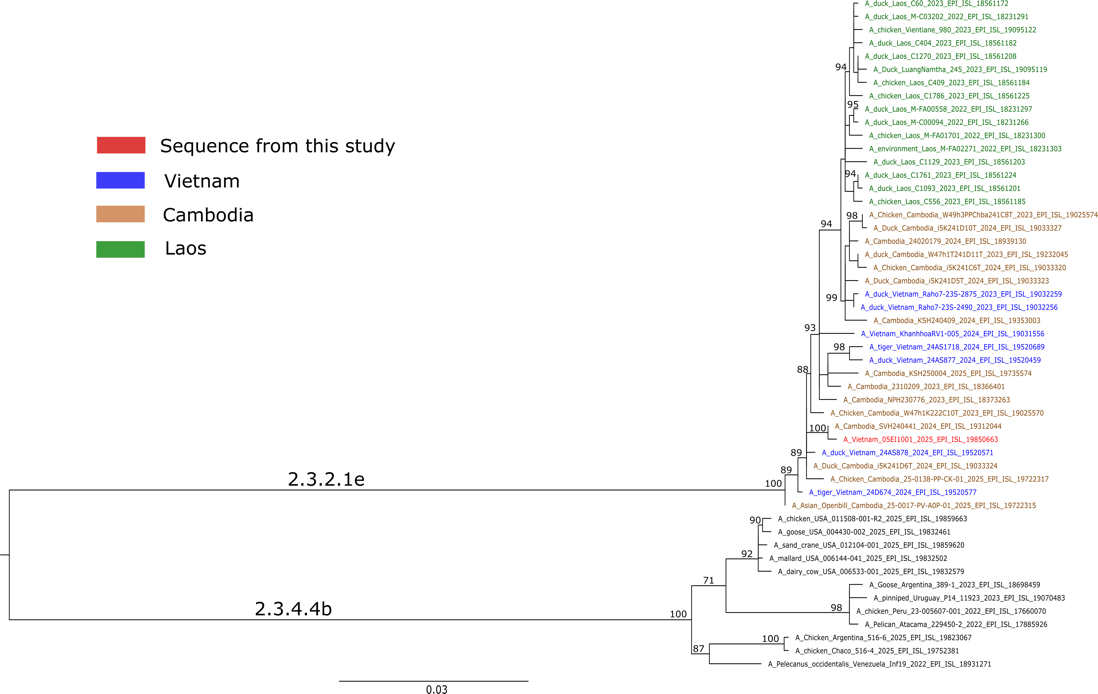


**E**


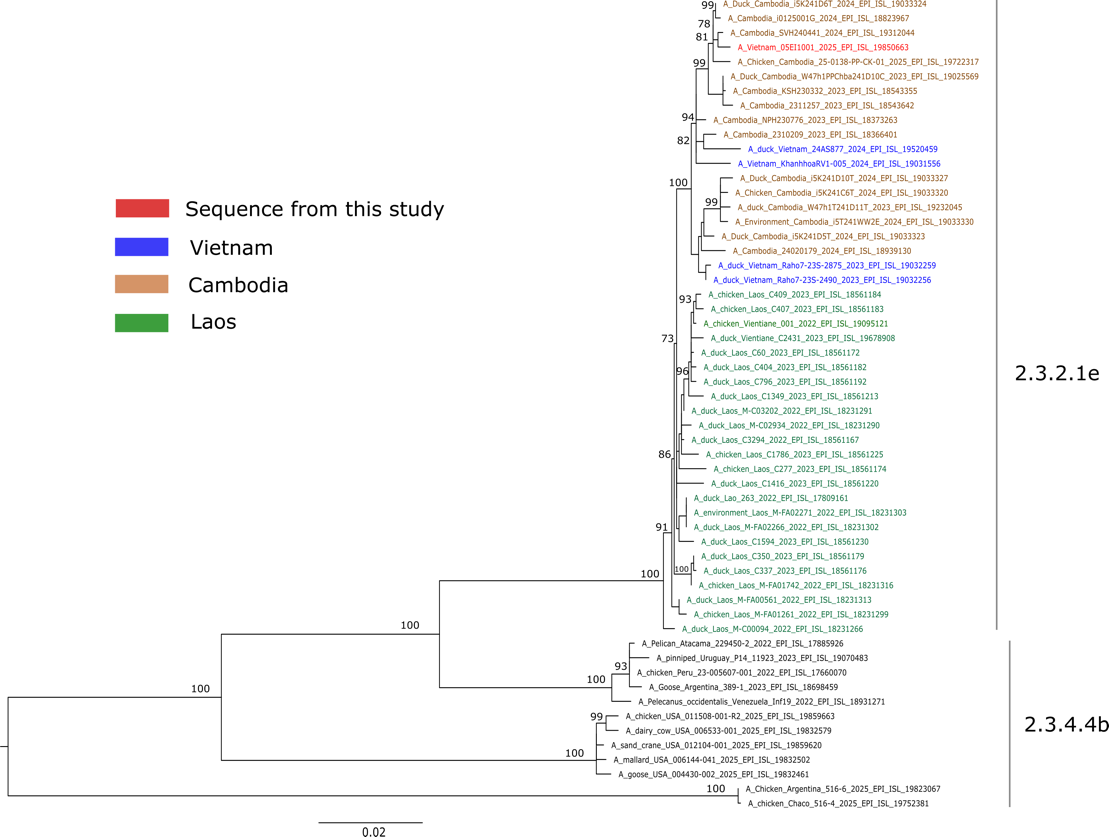


**F**


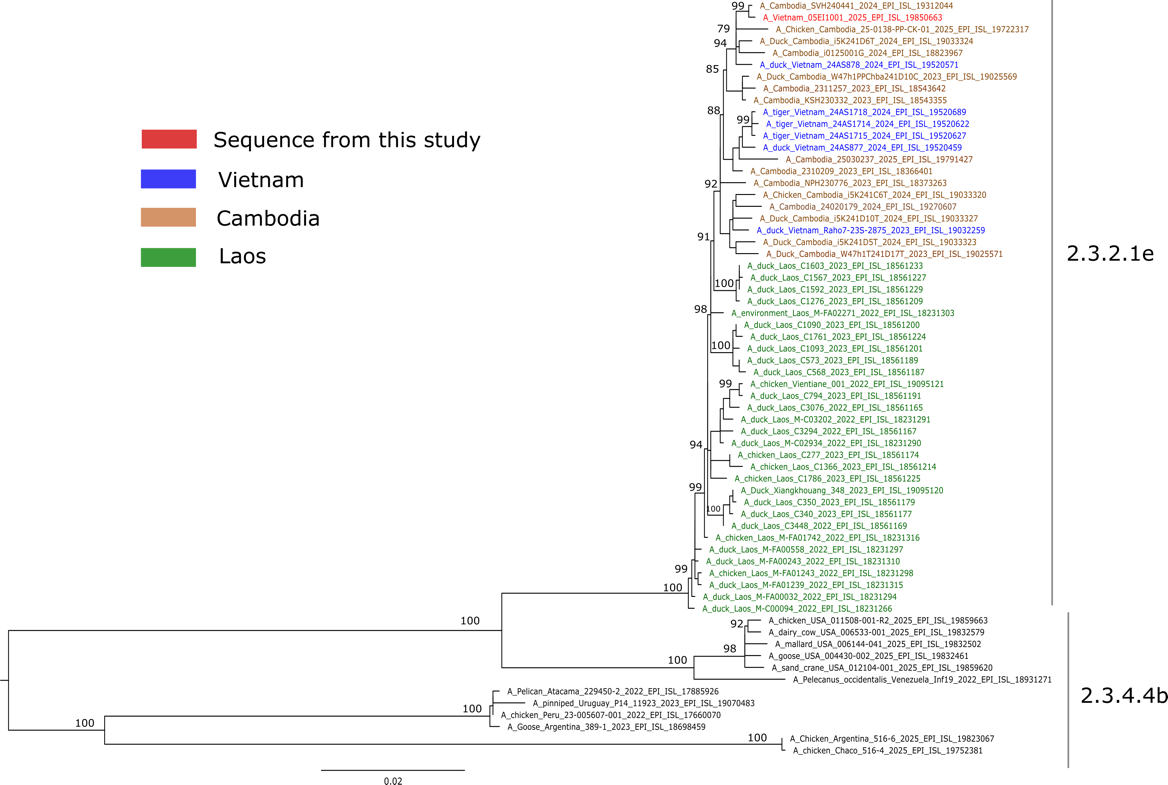


**G**


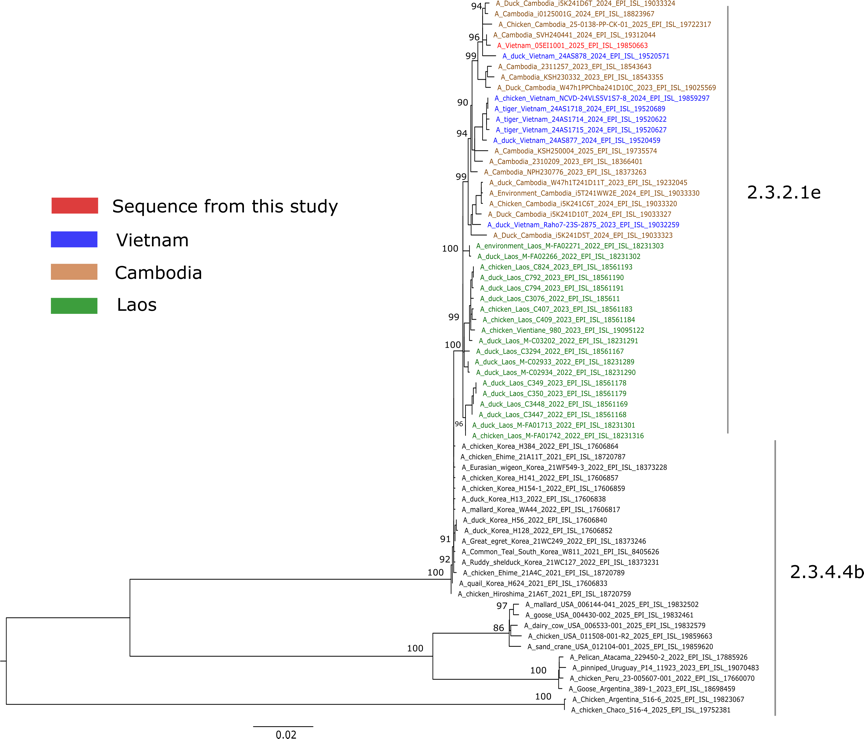


**Figure S3:** Reconstructed ML-trees of gene-segment sequences obtained from this study and representative the corresponding gene-segment sequences of clades 2.3.2.1e and 2.3.4.4b, A)) NA, B) MP, C) NP, D) NS, E) PA, F) PB1 and G) PB2.

**Additional Discussion**

According to the World Health Organization (WHO), from 2003 to 1 July 2025, sporadic A(H5N1) virus cases have been reported in more than 25 countries, resulting in 986 human infections, including 473 fatal (case-fatality rate (CFR): 48.0 %)[13]. Of the 986 reported cases, 129 (13.1%) came from Vietnam and 83 (8.4%) from Cambodia. Notably, both countries underwent a period of ~10 years with no reported human infections until 2023. Since then, there has been an increase in cases (n=27, 12 deaths, CFR: 44.4%) reported in Cambodia[13, 14], and two cases, one of which was fatal reported in Vietnam. The clinical presentations of the recently reported cases in Cambodia have not been fully described. However, preliminary sequence analysis suggested the responsible viruses of the most recent human infections in Cambodia also belonged to the novel reassortant clade 2.3.2.1e, and the reported cases were associated with severe acute respiratory infection [15].

To date there have been only seven reported human cases of A(H5N1) virus infection associated with CNS infection, including 5 children (including 1 from the present study) and 2 adults [16-22]. During the 2004 A(H5N1) virus outbreaks in Vietnam, which involved a total of 47 cases, all patients exhibited severe respiratory illness except for two fatal cases in children who experienced diarrhoea followed by coma[18]. Whether children are more vulnerable to severe disease (including CNS involvement) than adults should be investigated. Of note, prior infection with seasonal influenza viruses or annual influenza vaccination, which is not common practice in Vietnam, might confer cross reactivity against A(H5N1) virus[23]. In this context, as CSF penetration of oseltamivir is low[1], early hospital admission, access to supportive, or a combination of factors (including route of exposure) may explain the clinical consequences and outcome of our patient.

**REFERENCES**

1. Jhee, S.S., et al., *Low penetration of oseltamivir and its carboxylate into cerebrospinal fluid in healthy Japanese and Caucasian volunteers.* Antimicrob Agents Chemother, 2008. **52**(10): p. 3687-93.

2. Phung, N.T.N., et al., *Naegleria fowleri: Portrait of a Cerebral Killer.* Diagnostics (Basel), 2025. **15**(1).

3. Deregt, D., et al., *A microsphere immunoassay for detection of antibodies to avian influenza virus.* J Virol Methods, 2006. **137**(1): p. 88-94.

4. Jia, J.Z., et al., *Influenza antibody breadth and effector functions are immune correlates from acquisition of pandemic infection of children.* Nat Commun, 2024. **15**(1): p. 3210.

5. Li, Z.N., et al., *Novel multiplex assay platforms to detect influenza A hemagglutinin subtype-specific antibody responses for high-throughput and in-field applications.* Influenza Other Respir Viruses, 2017. **11**(3): p. 289-297.

6. Thanh, T.T., et al., *Value of lipocalin 2 as a potential biomarker for bacterial meningitis.* Clin Microbiol Infect, 2020. **27**(5): p. 724-30.

7. Chau, N.V.V., et al., *Immunogenicity of Oxford-AstraZeneca COVID-19 Vaccine in Vietnamese Health-Care Workers.* Am J Trop Med Hyg, 2022. **106**(2): p. 556-561.

8. Zhou, B., et al., *Single-reaction genomic amplification accelerates sequencing and vaccine production for classical and Swine origin human influenza a viruses.* J Virol, 2009. **83**(19): p. 10309-13.

9. Shepard, S.S., et al., *Viral deep sequencing needs an adaptive approach: IRMA, the iterative refinement meta-assembler.* BMC Genomics, 2016. **17**(1): p. 708.

10. Nguyen, L.T., et al., *IQ-TREE: a fast and effective stochastic algorithm for estimating maximum-likelihood phylogenies.* Mol Biol Evol, 2015. **32**(1): p. 268-74.

11. Giussani, E., et al., *FluMut: a tool for mutation surveillance in highly pathogenic H5N1 genomes.* Virus Evol, 2025. **11**(1): p. veaf011.

12. Perez-Sautu, U., et al., *Y155H amino acid substitution in influenza A(H1N1)pdm09 viruses does not confer a phenotype of reduced susceptibility to neuraminidase inhibitors.* Euro Surveill, 2014. **19**(27): p. 14-20.

13. Organization, W.H., *Disease Outbreak News; Avian Influenza A (H5N1) in Cambodia Available at:* [*https://www.who.int/emergencies/disease-outbreak-news/item/2024-DON575*](https://www.who.int/emergencies/disease-outbreak-news/item/2024-DON575)*.* 5 July 2025.

14. Siegers, J.Y., et al., *Emergence of a Novel Reassortant Clade 2.3.2.1c Avian Influenza A/H5N1 Virus Associated with Human Cases in Cambodia.* medRxiv, 2025.

15. Davis, W.W., et al., *Detecting Influenza A(H5N1) Viruses through Severe Acute Respiratory Infection Surveillance, Cambodia.* Emerg Infect Dis, 2026. **32**(3).

16. Mak, G.C.K., et al., *Influenza A(H5N1) Virus Infection in a Child With Encephalitis Complicated by Obstructive Hydrocephalus.* Clin Infect Dis, 2018. **66**(1): p. 136-139.

17. Zhang, L., et al., *Clinical features of the first critical case of acute encephalitis caused by the avian influenza A (H5N6) virus.* Emerg Microbes Infect, 2022. **11**(1): p. 2437-2446.

18. de Jong, M.D., et al., *Fatal avian influenza A (H5N1) in a child presenting with diarrhea followed by coma.* N Engl J Med, 2005. **352**(7): p. 686-91.

19. Chokephaibulkit, K., et al., *A child with avian influenza A (H5N1) infection.* Pediatr Infect Dis J, 2005. **24**(2): p. 162-6.

20. Gao, R., et al., *A systematic molecular pathology study of a laboratory confirmed H5N1 human case.* PLoS One, 2010. **5**(10): p. e13315.

21. Gu, J., et al., *H5N1 infection of the respiratory tract and beyond: a molecular pathology study.* Lancet, 2007. **370**(9593): p. 1137-45.

22. Rajabali, N., et al., *Avian influenza A (H5N1) infection with respiratory failure and meningoencephalitis in a Canadian traveller.* Can J Infect Dis Med Microbiol, 2015. **26**(4): p. 221-3.

23. Sidney, J., et al., *Targets of influenza human T-cell response are mostly conserved in H5N1.* mBio, 2025. **16**(2): p. e0347924.
